# Non-reassuring foetal status and neonatal irritability in the Japan Environment and Children’s Study: A cohort study

**DOI:** 10.1038/s41598-018-34231-y

**Published:** 2018-10-26

**Authors:** Seiichi Morokuma, Takehiro Michikawa, Kiyoko Kato, Masafumi Sanefuji, Eiji Shibata, Mayumi Tsuji, Ayako Senju, Toshihiro Kawamoto, Shouichi Ohga, Koichi Kusuhara

**Affiliations:** 1 Research Center for Environmental and Developmental Medical Sciences, Kyushu University, Fukuoka, Japan; 2 Department of Health Sciences, Graduate School of Medical Sciences, Kyushu University, Fukuoka, Japan; 3 Environmental Epidemiology Section, Centre for Health and Environmental Risk Research, National Institute for Environmental Studies, Tsukuba, Ibaraki Japan; 4 Department of Obstetrics and Gynecology, Graduate School of Medical Sciences, Kyushu University, Fukuoka, Japan; 5 Department of Pediatrics, Graduate School of Medical Sciences, Kyushu University, Fukuoka, Japan; 6 Japan Environment and Children’s Study, UOEH Subunit Center, University of Occupational and Environmental Health, Kitakyushu, Fukuoka Japan; 7 Department of Obstetrics and Gynecology, School of Medicine, University of Occupational and Environmental Health, Kitakyushu, Fukuoka Japan; 8 Department of Environmental Health, School of Medicine, University of Occupational and Environmental Health, Kitakyushu, Fukuoka Japan; 9 Department of Pediatrics, School of Medicine, University of Occupational and Environmental Health, Kitakyushu, Japan

## Abstract

The aim of this study was to investigate whether non-reassuring foetal status (NRFS) affected an infant’s temperament, or if the temperament formed prenatally resulted in an excessive heart rate reaction that was diagnosed as NRFS. We examined the correlation between NRFS and difficulty in holding a baby, and the amount of crying in the one month after birth, which was considered an indicator of the newborn’s temperament. We divided the cases with NRFS into positive NRFS and false positive NRFS. NRFS was associated with bad mood, frequent crying for a long duration, and intense crying. After adjustment for other covariates, NRFS was associated with bad mood (odds ratio, OR = 1.15, 95% confidence interval, CI = 1.00–1.33), and intense crying (1.12, 1.02–1.24). In the multi-variable model, positive and false positive NRFS were not clearly associated with neonatal irritability. When stratified by parity, NRFS and false positive NRFS were likely to be positively associated with neonatal irritability in parous women. The clear association between NRFS and intense crying was observed in parous women (multi-variable adjusted OR = 1.46, 95% CI = 1.16–1.83), but not in nulliparae (1.01, 0.91–1.12) (p for effect modification <0.01). Similarly, increased odds of intense crying associated with false positive NRFS were only found in parous women (multi-variable adjusted OR = 1.40, 95% CI = 1.09–1.81) (p for effect modification = 0.03). There was no association observed between positive NRFS and irritability; therefore, NRFS has no effect on an infant’s temperament.

## Introduction

Foetal distress during labour is a risk factor for developmental disorders, including autism^1,2^. It has also been reported that changes in the temperament of babies with diseases related to neuropsychiatric development can be seen in the neonatal and infant stages^3^. However, there are almost no reports on the association between foetal distress and infant temperament. Irritability is an important element of temperament. It can negatively affect parent-child relationships, which in turn can lead to child abuse. Furthermore, it affects the interactions between the mother and child as well as the child’s mental health.

To the best of our knowledge, only one study has investigated the relationship between postnatal irritability and changes in foetal heart rate during labour that was used to diagnose foetal distress. The study reported a correlation between reduced heart rate during labour and the proportion of crying as an indication of irritability up to day 5 after birth. However, it is unknown whether this reaction was due to hypoxia or acidaemia during labour, or it was simply caused by the predisposition of the neonate^4^.

Regarding the possibility that an infant’s personality is already formed in infancy, there are reports that heart rate reactivity in infancy is related to postnatal temperamental reactivity, and the characteristics of an infant’s behaviour represent the individual differences in reactivity during infancy^5,6^. If we presume that an infant’s temperament is already formed before birth, then their heart rate reactivity to the stress of labour may also differ.

Either way, in clinical practice in obstetrics and gynaecology, the well-being of the foetus is evaluated by monitoring the foetal heart rate. When abnormalities in foetal heart rate are observed, which are usually a result of hypoxia and/or acidaemia, the well-being of the foetus cannot be ascertained. However, even if there is an abnormality of the foetal heart rate, and a diagnosis of non-reassuring foetal status (NRFS) is made, normal umbilical cord arterial blood pH and Apgar scores are noted after birth. Therefore, NRFS false positives are thought to be associated with an increase in the number of Caesarean deliveries^7–9^. However, the cause of NRFS false positives is unknown^10,11^. Furthermore, the term ‘foetal distress’, an inaccurate and non-specific term, does not reflect the reality; therefore, this term is now being replaced with the term non-reassuring foetal status (NRFS)^12^. In this article, examples that were previously described as foetal distress are called NRFS.

Cases with foetal heart rate abnormalities that satisfied the criteria of an umbilical cord arterial blood pH ≥ 7.2 and an Apgar score ≥7 at 5 minutes were classified as NRFS false positive^13,14^. We believe that foetuses with these features and false positive NRFS had an overreaction to labour stress. Given that the amount of crying is said to be related to the behavioural characteristics of the newborn^15,16^, and the difficulty in holding a baby is said to be related to developmental characteristics^17^, we examined the correlation between NRFS and the difficulty in holding a baby and the amount of crying in the one month after birth that was considered the temperament of the newborn.

The aim of this study was to ascertain if NRFS affected an infant’s temperament, or if the temperament formed prenatally generated an excessive heart rate reaction diagnosed as NRFS.

## Methods

### Study participants

The Japan Environment and Children’s Study (JECS), an on-going large-scale birth cohort study, enrolled women as early in pregnancy as possible. The protocol of JECS is described elsewhere along with a discussion on its population representativeness^18,19^.

The JECS protocol was approved by the Japan Ministry of the Environment’s Institutional Review Board on Epidemiological Studies, and the Ethics Committees of all participating institutions. JECS protocol was conducted in accordance with the Helsinki Declaration and other nationally valid regulations, and written informed consent was obtained from each participant.

Between 2011–2014, a total of 103,099 pregnancies from fifteen Regional Centres located throughout Japan (Hokkaido, Miyagi, Fukushima, Chiba, Kanagawa, Koshin, Toyama, Aichi, Kyoto, Osaka, Hyogo, Tottori, Kochi, Fukuoka, and South Kyushu/Okinawa) were identified. Of the 103,099 pregnancies, only 97,454 unique mothers were included. Of these, we further excluded 27,129 women due to the following reasons (Supplementary Figure): multiple pregnancies (n = 949); miscarriages or stillbirths or no delivery records (n = 3,709); preterm (<37 weeks of gestation) or post-term (>41 weeks) deliveries (n = 4,586); missing information on maternal age at delivery, umbilical cord blood pH, or Apgar scores at five minutes (n = 16,351); and no responses to the three irritability questions in the maternal questionnaire at one month after delivery (n = 1,534). The remaining 70,325 participants were included in our final analyses.

### Non-reassuring foetal status (NRFS)

In the present study, we defined NRFS as that diagnosed by an obstetrician, which was obtained from the medical record transcriptions at birth^20^. In Japan, when abnormalities in foetal heart rate are observed, obstetricians diagnose the cases using intrapartum management guidelines based on the foetal heart rate pattern classification^20^. That is, the combination of the various components of the FHR patterns serves to estimate the degree of risk for the foetus, which is then classified into five levels. The five level categories are the same as those used in countries such as the United States^21^. Those with high risk levels of 3–5 are diagnosed with NRFS. However, in the cohort data, there was no information on the five levels, and only information on the presence or absence of NRFS was available. Of all the infants with NRFS, infants with an umbilical cord blood pH <7.2 and an Apgar score of <7 at 5 minutes after birth were categorised as having positive NRFS, and infants with an umbilical cord arterial blood pH ≥7.2 and an Apgar score ≥7 at 5 minutes were categorised as having false positive NRFS.

### Infant irritability

A self-administered questionnaire was distributed to mothers who visited for a health check-up at one month after delivery by research coordinators or via mail. The questionnaire included three questions about a baby’s irritability. The first question was “When you hold your baby, how often do you feel difficulty to hold your baby due to his/her fretting, crying or throwing his/her head back?” with options of “often”, “sometimes”, “seldom”, and “none”, and those who answered “often” were categorised as ‘bad’ mood. The second question was “How often and long does your baby cry?” with options of “quite often and long”, “sometimes and shortly”, and “seldom and hardly”, and those who answered as “quite often and long” were categorised as ‘frequent crying for a long duration’. The third question was “Does your baby cry very hard sometimes no matter what you do to stop him/her?” with options of “yes” and “no”, and those who answered as “yes” were categorised as ‘intense crying’.

### Covariates

Maternal characteristics, including maternal age at delivery, smoking habits, alcohol consumption, educational background, household income, parity, and infertility treatment, were collected via self-administered questionnaires and/or medical record during the pregnancy. Also, delivery and birth record transcripts were obtained to gather information about the gestational age at delivery, type of delivery, infant sex, and birth weight. In this study, small for gestational age (SGA) was defined as birth weight <10th percentile of birth weight standards by gestational age, sex, and parity for Japanese neonates^22^. Postpartum depressive symptoms were assessed using the Edinburgh Postnatal Depression Scale (EPDS) included in the questionnaire at one month after delivery^23^. According to previous studies, participants with a score of 9 or more were categorised as having depressive symptoms^24^.

### Statistical analyses

Multilevel logistic regression analyses were used to explore the association between NRFS and neonatal irritability, considering that mothers were nested in each Regional Centres, and to estimate the odds ratios (ORs) of neonatal irritability and 95% confidence intervals (CIs), without NRFS as a reference. We initially adjusted for maternal age at delivery, and then further adjusted for educational background (<10, 10–12, 13–16, ≥17 years), household income (<2, 2 to <4, 4 to <6, 6 to 8, 8 to <10, ≥10 million Japanese-yen), smoking habits (never smoked, ex-smokers who quit before pregnancy, smokers during early pregnancy), alcohol consumption (never drank, ex-drinkers who quit before pregnancy, drinkers during early pregnancy), gestational age at birth (37–38, 39–40, and 41 weeks of gestation), SGA (yes, no), infant sex (boys, girls), parity (0, ≥1), infertility treatment (yes, no), type of delivery (vaginal, Caesarean), and postpartum depressive symptoms (yes, no).

When the frequencies of NRFS were affected by certain background factors (except clinical known factors), we performed stratified analyses. Effect modification of the association between NRFS and neonatal irritability according to such factors was evaluated by a likelihood ratio test. This study used the dataset jecs-ag-20160424, which was released in June 2016, and revised in October 2016. Stata version 14 (StataCorp LP, College Station, Texas, USA) was used for all analyses.

## Results

The mean maternal age at delivery was 31.2 (standard deviation, SD = 5.1) years in this study population. The baseline characteristics in those with or without NRFS are shown in Table 1. Naturally, the distribution of the type of delivery, gestational age, and SGA differed by the presence or absence of NRFS. Mothers categorised as NRFS tended to be nulliparous and delivered boys. The frequency of NRFS was 3.9%. Among the cases of NRFS, 3.3% were positive NRFS, 83.3% were false positive NRFS and 13.4% did not belong to any of these groups.

**Table 1:** Baseline characteristics of participants in the Japan Environment and Children’s Study (2011–2014).

Non-Reassuring Foetal Status	No (n = 67,559)		Yes (n = 2,766)	
	n*	%	n*	%
**Maternal characteristics**				
Age at delivery (years)				
<25	6,638	9.8	248	9.0
25–29	18,611	27.6	715	25.9
30–34	23,870	35.3	990	35.8
≥35	18,440	27.3	813	29.4
Educational background (years)				
<10	3,042	4.6	114	4.2
10–12	21,053	31.6	780	28.5
13–16	41,680	62.5	1,803	65.8
≥17	959	1.4	42	1.5
Household income (million Japanese-yen/year)				
<2	3,449	5.5	143	5.6
2 to <4	21,449	34.5	838	32.7
4 to <6	20,542	33.0	826	32.2
6 to <8	9,995	16.1	457	17.8
8 to <10	4,172	6.7	194	7.6
≥10	2,658	4.3	109	4.3
Smoking habits				
Never smoked	39,387	58.5	1,656	60.0
Ex-smokers who quit before pregnancy	15,891	23.6	572	20.7
Smokers during early pregnancy	12,033	17.9	531	19.3
Alcohol consumption				
Never drank	23,363	34.7	896	32.5
Ex-drinkers who quit before pregnancy	12,508	18.6	454	16.5
Drinkers during early pregnancy	31,509	46.8	1,410	51.1
Parity				
0	28,677	42.6	2,081	75.4
≥1	38,680	57.4	678	24.6
Infertility treatment				
No	63,131	93.5	2,441	88.3
Yes	4,405	6.5	324	11.7
Type of delivery				
Vaginal	56,584	83.8	1,340	48.5
Caesarean	10,975	16.3	1,426	51.6
Gestational age (weeks)				
37–38	22,478	33.3	640	23.1
39–40	38,691	57.3	1,621	58.6
41	6,390	9.5	505	18.3
Postpartum depressive symptoms at 1 month after delivery assessed by the Edinburgh Postnatal Depression Scale				
No (score <8)	57,076	85.8	2,251	82.4
Depressive (score ≥9)	9,483	14.3	482	17.6
Infant characteristics				
Birth weight				
mean (standard deviation) (g)	3,065 (364)		2,978 (414)	
Small for gestational age	4,702	7.0	469	17.0
Infant sex				
Boys	34,224	50.7	1,606	58.1
Girls	33,335	49.3	1,160	41.9

*Adjusted for maternal age, educational background, household income, smoking habits, alcohol consumption, gestational age at birth, small for gestational age, infant sex, parity, infertility treatment, type of delivery, and postpartum depressive symptoms *Numbers in subgroups do not equal overall number because of missing data.

Table 2 shows the ORs for NRFS and neonatal irritability. In the maternal age-adjusted model, we observed the association of NRFS with a bad mood, frequent crying for a long duration, and intense crying. After adjustment for other covariates, NRFS was associated with a bad mood (OR = 1.15, 95% CI = 1.00–1.33), and intense crying (1.12, 1.02–1.24), although point estimates of OR were attenuated. In the multi-variable model, positive and false positive NRFS were not clearly associated with neonatal irritability.

**Table 2:** Association between non-reassuring foetal status (NRFS) and neonatal irritability.

	n	Number of outcomes	Frequency of outcome (%)	Maternal age-adjusted model		Multi-variable model*	
				OR	95% CI	OR	95% CI
**NRFS**							
Bad mood							
no NRFS	67,438	4,108	6.1	Reference		Reference	
NRFS	2,764	287	10.4	1.79	(1.58–2.03)	1.15	(1.00–1.33)
Frequent crying for a long duration							
no NRFS	67,294	11,501	17.1	Reference		Reference	
NRFS	2,755	642	23.3	1.46	(1.34–1.60)	1.08	(0.98–1.20)
Intense crying							
no NRFS	67,324	12,931	19.2	Reference		Reference	
NRFS	2,760	774	28.0	1.65	(1.51–1.80)	1.12	(1.02–1.24)
**Positive NRFS**							
Bad mood							
no NRFS	67,438	4,108	6.1	Reference		Reference	
NRFS	89	10	11.2	1.95	(1.01–3.77)	1.40	(0.71–2.76)
Frequent crying for a long duration							
no NRFS	67,294	11,501	17.1	Reference		Reference	
NRFS	90	26	28.9	1.98	(1.26–3.13)	1.54	(0.94–2.52)
Intense crying							
no NRFS	67,324	12,931	19.2	Reference		Reference	
NRFS	90	19	21.1	1.14	(0.69–1.89)	0.88	(0.52–1.50)
**False positive NRFS**							
Bad mood							
no NRFS	67,438	4,108	6.1	Reference		Reference	
NRFS	2,303	232	10.1	1.73	(1.50–1.99)	1.10	(0.94–1.29)
Frequent crying for a long duration							
no NRFS	67,294	11,501	17.1	Reference		Reference	
NRFS	2,296	522	22.7	1.42	(1.28–1.57)	1.05	(0.94–1.17)
Intense crying							
no NRFS	67,324	12,931	19.2	Reference		Reference	
NRFS	2,298	643	28.0	1.64	(1.50–1.80)	1.10	(0.99–1.22)

CI = confidence interval, OR = odds ratio.

When we stratified by parity, NRFS and false positive NRFS were likely to be positively associated with neonatal irritability in parous women (Table 3). The clear association between NRFS and intense crying was observed in parous women (multi-variable adjusted OR = 1.46, 95% CI = 1.16–1.83), but not in nulliparous women (1.01, 0.91–1.12) (p for effect modification <0.01). Similarly, increased odds of intense crying associated with false positive NRFS were only found in parous women (multi-variable adjusted OR = 1.40, 95% CI = 1.09–1.81) (p for effect modification = 0.03). The number of cases of positive NRFS was small; therefore, we did not perform stratified analysis in these cases. Effect modification of the association between NRFS and neonatal irritability was not observed with the sex of the infant (Supplementary Table).

**Table 3:** Association between non-reassuring foetal status (NRFS) and neonatal irritability stratified by parity.

	Nulliparae					Parous women					p for effect modification**
	n	Number of outcomes	Frequency of outcome (%)	Adjusted OR*	95% CI	n	Number of outcomes	Frequency of outcome (%)	Adjusted OR*	95% CI	
**NRFS**											
Bad mood											
no NRFS	28,633	3,155	11.0	Reference		38,603	934	2.4	Reference		0.24
NRFS	2,079	262	12.6	1.08	(0.93–1.25)	678	24	3.5	1.48	(0.96–2.30)	
Frequent crying for a long duration											
no NRFS	28,548	7,053	24.7	Reference		38,547	4,407	11.4	Reference		0.17
NRFS	2,073	542	26.2	1.01	(0.90–1.13)	675	99	14.7	1.22	(0.96–1.54)	
Intense crying											
no NRFS	28,586	8,895	31.1	Reference		38,537	3,996	10.4	Reference		<0.01
NRFS	2,076	673	32.4	1.01	(0.91–1.12)	677	99	14.6	1.46	(1.16–1.83)	
**False positive NRFS**											
Bad mood											
no NRFS	28,633	3,155	11.0	Reference		38,603	934	2.4	Reference		0.50
NRFS	1,730	213	12.3	1.04	(0.88–1.23)	568	18	3.2	1.32	(0.79–2.19)	
Frequent crying for a long duration											
no NRFS	28,548	7,053	24.7	Reference		38,547	4,407	11.4	Reference		0.26
NRFS	1,725	440	25.5	0.98	(0.86–1.11)	566	81	14.3	1.17	(0.90–1.52)	
Intense crying											
no NRFS	28,586	8,895	31.1	Reference		38,537	3,996	10.4	Reference		0.03
NRFS	1,726	560	32.4	0.99	(0.88–1.11)	567	81	14.3	1.40	(1.09–1.81)	

CI = confidence interval, OR = odds ratio.

^*^Adjusted for maternal age, educational background, household income, smoking habits, alcohol consumption, gestational age at birth, small for gestational age, infant sex, infertility treatment, type of delivery, and postpartum depressive symptoms.

^**^Effect modification according to parity was evaluated by a likelihood ratio test.

## Discussion

Although all cases of NRFS were associated with a bad mood, and intense crying, cases of positive and false positive NRFS were not clearly associated with infant irritability. However, the clear association between false positive NRFS and intense crying was observed in parous women. Therefore, the events during labour did not affect the irritability of the newborns, and, thus, the irritability had formed prenatally.

Some reports have stated that the intrauterine environment affects an infant’s mental development^25–28^, while others have stated that it affects the temperament of neonates^29^. Furthermore, recently there have been reports on the link between the autonomic nervous system reactivity and temperament; people with small fluctuations in their heart rate during the resting phase have excessive circulation reactions–the smaller the fluctuation, the less able they are to control their emotions, and their emotional reactions are greater–and they are less able to withstand significant stress^30–32^.

Factors such as congenital malformations, infections, and cigarette smoking have been linked to false positive NRFS; furthermore, there are reports of a high rate of false positive NRFS even when the foetus’ well-being has been maintained^33–35^. Despite NRFS resulting in emergency Caesarean section deliveries in foetuses with cardiac malformations, many of them do not have acidosis^36^. Foetal heart rate patterns are mainly produced by the autonomic nervous system and the cardiovascular centre in the brain stem^10,11^. We believe that the false positive NRFS may be caused by several factors that can affect these systems in the absence of hypoxia and/or acidaemia.

Recently there has been an increasing number of reports about the effect of maternal inflammation and immune activity on the neuropsychiatric development of a child^37–39^. We previously clarified that air pollution exposure during early pregnancy, which results in systemic inflammation, is related to false positive NRFS^40^. The intrauterine environment affects the foetus, and it may affect the irritability of the newborn; therefore, so we presume that false positive NRFS also incorporate factors related to the infant’s temperament.

One of the limitations of this study was that the cohort data had no information on the NRFS 5-grade classification; only data on the presence or absence of NRFS after diagnosis by an obstetrician was available. Thus, we could not perform analyses for the FHR pattern risk level in this study. However, since obstetricians diagnosed the patients using intrapartum management guidelines based on the foetal heart rate pattern classification, at least the diagnosis of NRFS can be considered accurate. We expect that analyses based on the FHR pattern risk levels will be conducted in the future.

In addition, another limitation of this study is that the information was collected from the mothers via a questionnaire. The infants were not directly examined, which could possibly result in a bias. However, the strength of this study is in its large sample size. Therefore, we believe that a certain trend can be detected; namely, the differences in experiences of the mothers depending on the birth order of the child may have affected the outcome. Therefore, we conducted our investigation after classifying the mothers based on parity^41–43^. An association was found between false positive NRFS and intense crying only in multipara women. With the first child, the mother has little childrearing experience, she is unable to make a comparison, which may have suppressed the distribution spread. Either way, there was no correlation seen between positive NRFS and irritability. Therefore, we believe that NRFS has no effect on an infant’s temperament.

## Electronic supplementary material

Supplementary figure & table
