# Supplementary material for: Non-reassuring foetal status and neonatal irritability in the Japan Environment and Children’s Study: A cohort study
Source: Sci Rep. 2018 Oct 26;8:15853. doi: 10.1038/s41598-018-34231-y (PMC6203769; doi:10.1038/s41598-018-34231-y)
Supplement: Supplementary file 1 — Supplementary figure & table [file 41598_2018_34231_MOESM1_ESM.pdf]

Title:

Non-reassuring foetal status and neonatal irritability in the Japan Environment and Children's Study: A cohort study

Authors:

Seiichi Morokuma[a, b*], Takehiro Michikawa[c*], Kiyoko Kato[a, d], Masafumi Sanefuji[a, e], Eiji Shibata[f, g], Mayumi Tsuji[h], Ayako Senju[f, i], Toshihiro Kawamoto[f, h], Shouichi Ohga[a, e], Koichi Kusuhara[f, i]

[a] Research Center for Environmental and Developmental Medical Sciences, Kyushu University, Fukuoka, Japan

[b] Department of Health Sciences, Graduate School of Medical Sciences, Kyushu University, Fukuoka, Japan

[c] Environmental Epidemiology Section, Centre for Health and Environmental Risk Research, National Institute for Environmental Studies, Tsukuba, Ibaraki, Japan

[d] Department of Obstetrics and Gynecology, Graduate School of Medical Sciences, Kyushu University, Fukuoka, Japan

[e] Department of Pediatrics, Graduate School of Medical Sciences, Kyushu University, Fukuoka, Japan

[f] Japan Environment and Children's Study, UOEH Subunit Center, University of Occupational and Environmental Health, Kitakyushu, Fukuoka, Japan

[g] Department of Obstetrics and Gynecology, School of Medicine, University of Occupational and Environmental Health, Kitakyushu, Fukuoka, Japan

[h] Department of Environmental Health, School of Medicine, University of Occupational and Environmental Health, Kitakyushu, Fukuoka, Japan

[i] Department of Pediatrics, School of Medicine, University of Occupational and Environmental Health, Kitakyushu, Japan

[*]These authors contributed equally to this work.

Corresponding author: Seiichi Morokuma, MD, PhD

Department of Obstetrics and Gynecology

Kyushu University Hospital, Kyushu University

3-1-1 Maidashi, Higashi-ku, Fukuoka 812-8582, Japan

Tel: +81-92-642-5105

Fax: +81-92-642-5105

E-mail: morokuma@med.kyushu-u.ac.jp

Supplementary figure.

Flow chart for recruitment of participants in this study

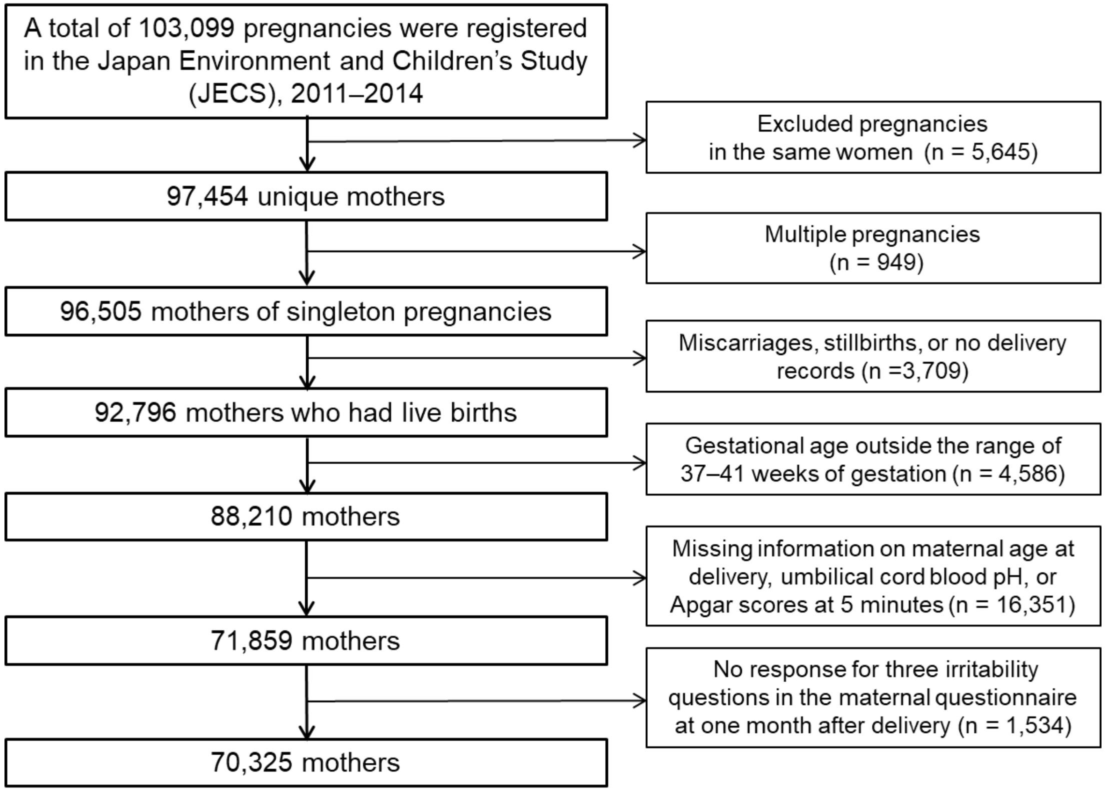

Supplementary Table. Association between non-reassuring fetal status (NRFS) and neonatal irritability stratified by infant sex

	Boys					Girls					
	n	Number of outcomes	Frequency of outcome (%)	Adjusted OR*	95% CI	n	Number of outcomes	Frequency of outcome (%)	Adjusted OR*	95% CI	p for effect modification**
NRFS											
Bad mood											
no NRFS	34,163	2,292	6.7	Reference		33,275	1,816	5.5	Reference		0.75
NRFS	1,605	177	11.0	1.15	(0.96–1.38)	1,159	110	9.5	1.16	(0.92–1.45)	
Frequent crying for a long duration											
no NRFS	34,101	6,222	18.3	Reference		33,193	5,279	15.9	Reference		0.48
NRFS	1,600	398	24.9	1.13	(0.99–1.29)	1,155	244	21.1	1.02	(0.87–1.20)	
Intense crying											
no NRFS	34,105	6,592	19.3	Reference		33,219	6,339	19.1	Reference		0.92
NRFS	1,603	445	27.8	1.13	(1.00–1.29)	1,157	329	28.4	1.12	(0.96–1.30)	
False positive NRFS											
Bad mood											
no NRFS	34,163	2,292	6.7	Reference		33,275	1,816	5.5	Reference		0.63
NRFS	1,319	140	10.6	1.08	(0.89–1.33)	984	92	9.4	1.14	(0.89–1.46)	
Frequent crying for a long duration											
no NRFS	34,105	6,222	18.3	Reference		33,193	5,279	15.9	Reference		0.64
NRFS	1,314	318	24.2	1.08	(0.94–1.25)	982	204	20.8	1.00	(0.84–1.19)	
Intense crying											
no NRFS	34,105	6,592	19.3	Reference		33,219	6,339	19.1	Reference		0.30
NRFS	1,316	355	27.0	1.06	(0.92–1.22)	982	288	29.3	1.16	(0.99–1.36)	

CI = confidence interval, OR = odds ratio

*Adjusted for maternal age, educational background, household income, smoking habits, alcohol consumption, gestational age at birth, small for gestational age, parity, infertility treatment, type of delivery, and postpartum depressive symptoms.

**Effect modification according to infant sex was evaluated by a likelihood ratio test.
